# Gene Array Studies in Renal Neoplasia

**DOI:** 10.1100/tsw.2006.109

**Published:** 2006-04-26

**Authors:** John P.T. Higgins

**Affiliations:** Department of Pathology, Stanford University School of Medicine, Stanford, CA 94305, USA

**Keywords:** kidney, immunohistochemistry, cDNA

## Abstract

Renal cell carcinoma (RCC) is comprised of several distinct histologic subtypes many of which have characteristic cytogenetic abnormalities. The molecular pathogenesis of some of these neoplasms is beginning to be elucidated. Yet renal cell carcinoma is often discovered at an advanced clinical stage and effective pharmacologic therapies for this disease remain to be discovered. For these reasons, renal cell carcinoma is ideally suited to the genome scale investigation made possible by DNA microarrays. A number of DNA array studies of renal cell carcinoma have been published. Renal cell carcinomas have also been studied by array based comparative genomic hybridization. The purpose of this review will be to summarize these studies, to compare the results of the different studies, and to suggest future areas of investigation with a particular emphasis on clinically relevant advances.

## INTRODUCTION

Renal cell carcinoma (RCC) is the ninth most common malignancy in the U.S. with 35,000 new cases and 12,000 deaths annually[1]. The incidence is increasing, which is due only in part to a higher rate of incidental detection[2]. Because of the anatomic location of the kidneys, renal neoplasms may attain a large size prior to detection and over one-quarter of patients present with metastatic disease. Yet the mainstay of treatment is surgical, with poor response rates for chemotherapy and radiotherapy. Clearly, additional insight into the pathogenesis of these neoplasms would be of potential benefit to those patients with advanced disease. Additionally, such insight might make early detection possible if serum markers of the disease can be identified.

The molecular genetic basis of clear cell RCC has been a topic of intense investigation and some important discoveries have been made. Patients with the von Hippel-Lindau syndrome develop multiple RCC. The tumor suppressor gene associated with this syndrome was discovered in 1993[3] and its involvement in hypoxia sensing has subsequently been characterized[4]. This gene is somatically mutated or hypermethylated in at least 60% of sporadic clear cell carcinomas[5,6]. The molecular genetic and clinical features of RCC have been recently reviewed[7]. It is clear that much remains to be learned and that studies on a genomic scale using microarrays may permit additional insights into the pathogenesis of this disease.

Adult renal neoplasms have undergone a revolution in classification over the past 30 years. The current WHO classification[8] recognizes five major types of adult renal neoplasm: clear cell carcinoma, papillary carcinoma, chromophobe carcinoma, collecting duct carcinoma, and oncocytoma. One of the seminal papers on grading of "renal cell carcinoma" and on which current grading schemes are based, that by Fuhrman and colleagues from 1982[9], did not subclassify the renal tumors and actually includes oncocytomas. Nevertheless, the clinical stratification made possible by this grading scheme is still of clinical value because approximately 70% of all adult renal tumors are of clear cell type. This preponderance of clear cell tumors must always be borne in mind since, even today, papers are published that refer simply to "renal cell carcinoma" without further characterization.

More than any other group of epithelial neoplasms, the classification of adult renal tumors has been driven by cytogenetic abnormalities[10,11,12]. This is true to the point that the finding of deletions of the short arm of chromosome 3 at the site of the von Hippel-Lindau gene is regarded as presumptive evidence of clear cell carcinoma, even if the cytologic features and growth pattern of the tumor are not typical of clear cell carcinoma[3,6,13,14]. Therefore, renal neoplasms seem ideally suited to benefit from the detailed molecular characterization made possible by DNA microarrays.

Gene microarray technology has been applied to all major classes of human neoplasia. These studies partly serve to underscore the effectiveness of tumor classification by conventional microscopy. However, unsupervised clustering techniques often suggest previously unrecognized classes of malignancy that often have clinical implications[15,16]. Furthermore, even when classification of neoplasms by microarray does not offer any further refinement to conventional classification, differential patterns of gene expression across the different classes of tumor may suggest particular genes that may be used to distinguish between classes in cases that show borderline morphologic features. The purpose of this review will be to summarize the finding of gene array studies of renal neoplasia and discuss some of the issues that may improve future investigation.

## GENE EXPRESSION STUDIES

### Renal Cell Carcinoma vs. Normal Kidney

Differences in gene expression between normal and neoplastic renal tissue represent a logical target for addressing the pathogenesis of RCC. Such studies may also permit detection of diagnostic biomarkers present in serum of patients with early RCC. Early gene microarray studies attempted to address expression differences between renal carcinoma cell lines and non-neoplastic renal tissue. Moch et al. compared the RCC-derived cell line, CRL-1933, with normal kidney tissue[17] and were able to identify vimentin expression in RCC. They subsequently confirmed this finding using tissue microarrays with paraffin-embedded renal carcinoma tissue.

Other investigators have noted that gene expression patterns are significantly altered in *ex vivo* cultured cells relative to their parent tumors[18] and have elected to study whole tissue. Boer et al.[19] studied 32 primary and 5 metastatic renal cell carcinomas using 31,500 element cDNA arrays. They found 1,738 genes that were differentially expressed in RCC vs. matched normal tissue. A number of genes that were highly expressed in tumors were involved in cell adhesion. These included fibronectin 1, collagen 4A, and laminin A4. Genes involved in nucleotide and nucleic acid metabolism were also represented among the up-regulated genes. In contrast, genes encoding transport proteins, and proteins involved in ion homeostasis, oxygen and radical metabolism, and electron transport were underexpressed. In a subsequent study[20], this group studied 112 RCC using 4,207 gene arrays that were custom made based partly on interesting genes discovered in the first study. They again found high expression in clear cell carcinoma of genes involved in angiogenesis, cell adhesion, and immune response and were able to identify a set of 32 markers genes that could be used to discriminate between clear cell, papillary, and chromophobe carcinomas. Because this group had excellent cytogenetic data from their patients, they were also able to correlate gene expression changes with karyotypic abnormalities. Furthermore, although their arrays were not designed for this purpose, they were able to detect gene expression patterns in the primary tumors that correlated with metastasis and outcome.

Other investigators have explored the general function of groups of genes that show altered expression and found similar results. Liou and colleagues studied six clear cell RCC tissue samples together with cell lines derived from clear cell RCC metastases and compared them to normal tissues matched to the clear cell RCC tissue samples[21]. These investigators also noted that cell adhesion genes tended to be up-regulated, whereas transport genes were down-regulated. Gieseg et al.[22] studied eight normal kidneys in comparison to nine clear cell carcinomas, two chromophobe carcinomas, one urothelial carcinoma, and one metanephric adenoma. They used a gene categorization system called Functional Taxonomy to discover that clear cell carcinomas showed relatively high expression of genes involved in signal transduction and cellular matrix organization and adhesion.

Using 60,000 element arrays, Skubitz and Skubitz compared clear cell RCC to diseased and normal kidney samples as well as to other epithelial malignancies[23]. They identified 151 genes and 115 Expressed sequence tag (ESTs) that were more than fourfold up-regulated in RCC relative to normal kidney, and 35 genes and 19 ESTs that were more than 10 times underexpressed in RCC vs. normal kidney. The up-regulated genes were further categorized into those that appeared specific for RCC, those that were common to RCC as well as to other malignancies and limited nonessential tissues, and those that were expressed in multiple tissues.

Jones et al.[24] compared normal kidney to $T_1$ clear cell carcinomas and not only identified differentially expressed genes, but also specifically focused on genes coding for secreted proteins and for cell surface receptors. Their rationale was that the former represent candidates for diagnostic serum markers, whereas the latter represent potential therapeutic targets.

Lenburg et al.[25] studied 18 RCC tissues and compared them to normal kidney samples using Affymetrix arrays and identify 1,234 genes that are differentially expressed in tumor vs. normal tissue and note that 800 of these had not been previously reported in earlier microarray studies. The authors used a gene-filtering method that stresses the elimination of poorly measured samples to improve the reliability of their samples. They performed a keyword analysis of the differentially expressed genes and discovered that genes associated with keywords such as hypoxia, angiogenesis, tumor necrosis factor, apoptosis, interferon, drug resistance, and metastasis were associated with the overexpressed genes.

Renal tumor differentiation relative to different segments of the normal nephron was explored by Schuetz and coworkers[26]. They found that clear cell and papillary RCC express genes typically expressed in the proximal nephron such as cubilin, megalin/low-density lipoprotein related protein 2, and α-methyl CoA recemase, whereas expression of distal nephron markers such as parvalbumin, chloride channels Ka and Kb, β-defensin 1, and claudin 7 characterized chromophobe RCC and oncocytoma.

## Renal Cell Carcinoma vs. Other Epithelial Malignancies

Molecular differences between renal cell and other carcinomas may shed light on the pathogenesis of these tumors, but also may be exploited for diagnostic purposes. A gene that is only expressed in RCC, but not in lung, breast, or prostate carcinoma, could be detected by immunohistochemistry in a liver metastasis of unknown primary. If the product of such a gene is secreted, then the possibility exists for serum detection of early tumors. Amatschek et al.[27] compared lung squamous and adenocarcinomas, breast carcinoma, and RCC using a PCR-based cDNA subtraction and cDNA microarray technique. These researchers found that RCC showed the most homogeneous pattern expression of the tumor types studied and identified hypoxia-inducible genes as distinguishing RCC from the other carcinomas.

## Class Distinction

Several studies have made it clear that unsupervised groupings of renal tumors based on gene expression patterns largely recapitulate classification based on morphologic parameters[19,26,28,29]. These studies are remarkable for the fact that a large range of platforms with different numbers and types of neoplasms show similar class groupings and relationships among different classes[22,28,29,30,31,32]. These studies show that the greatest dissimilarity in gene expression exists between clear cell carcinomas and all other neoplasms. Only rarely do clear cell tumors fail to group with other clear cell tumors and only rarely is a clear cell cluster infiltrated by nonclear cell tumors. Papillary carcinomas also show consistent patterns of gene expression, although the number of studied cases is fewer as would be expected. Another remarkably consistent finding is of similar gene expression patterns between oncocytoma and chromophobe carcinoma[24,26,28,29,31]. Although cases with morphologic overlap certainly exist, it is striking that even histologically distinct tumors may group together. As has been noted, this certainly would seem to support an interpretation that chromophobe carcinoma and oncocytoma are related neoplasms[31]. Finally, RCC with granular cytoplasm have proved controversial by molecular classification as well as by histology. The diagnostic category “granular RCC” was abandoned in the Heidelberg classification, with the justification being that tumors of different and recognizable type had since been described and, therefore, the term was nonspecific. Granular cytoplasm may be seen in chromophobe carcinoma or oncocytoma, but is also typical of some conventional RCC as well. In our series[28], we found that the conventional carcinomas with granular cytoplasm showed a markedly dissimilar pattern of expression from the conventional clear cell carcinomas and that they were heterogeneous, grouping with tumors of several other classes. Takahashi et al.[29] reported six cases that had originally been classified as “granular RCC” and found that they showed similar gene expression patterns to those tumors with which they would be grouped based on a rereview of the histology.

A directed effort at refining the classification of papillary RCC was undertaken by Yang et al.[33] who used expression analysis in order to identify two distinct types of papillary carcinoma. These were reconciled with two classes of papillary carcinoma recognized by histologic means[34]. The first molecular class of papillary carcinoma represented all of the type 1 carcinomas, but also included the type 2 carcinomas of low cytologic grade and those that were mixed type 1 and low-grade type 2. The second class was comprised of tumors that had high-grade type 2 morphology. The class 2 tumors had a poorer prognosis, but the molecular designation was not significantly independent of the conventional stratification based on grade and stage. The class 1 tumors were characterized by cytokeratin 7 expression, whereas the class 2 tumors typically expressed topoisomerase IIα.

## Prognostic Stratification

One principal goal of expression analysis is to identify genes or patterns of gene expression that permit a refined assessment of prognosis for affected patients. Takahashi et al. succeeded in identifying distinct patterns of gene expression between clear cell carcinomas with a good outcome and those that were associated with a poor outcome[35]. Using 22,000 element arrays, these investigators were able to stratify 29 clear cell carcinoma patients into 2 groups. The first group enjoyed a 100% 5-year survival and 88% of patients were free of metastasis at 5 years, whereas the second group had a 0% 5-year survival time and an average survival time of 25.4 months. Stratification could be accomplished using only 40 genes and ESTs (represented by 51 spots). This prognostic stratification based on gene expression profile was superior to that based on stage, but similar to that based on grade. The genes responsible for the stratification included those related to angiogenesis and matrix metalloproteinase activity.

Vasselli et al.[36] studied gene expression in the primary tumors of 58 patients who presented with stage IV RCC (51 clear cell, 6 papillary, and 1 undifferentiated) using 6,400 element cDNA arrays. They found a small difference in survival between the 2 principal groups identified by unsupervised hierarchical clustering. They then selected the 45 genes most strongly correlated with survival and showed that these genes could be used to stratify patients into longer and shorter survival groups. They found that the most strongly predictive individual gene, VCAM-1, could alone be used to stratify patient survival. Immunohistochemistry for VCAM-1 was performed on 9 patients, but was described as heterogeneous and does not appear to have been pursued further.

More recently, Zhao et al. surveyed 177 clear cell carcinomas and identified 5 distinct expression groups by unsupervised hierarchical clustering[37]. These subgroups had prognostic relevance independent of stage, grade, and performance status. Furthermore, one of their subgroups showed relatively lower expression of hypoxia-regulated genes, suggesting that these tumors may lack VHL mutations. The subgroup with the worst prognosis was characterized by high expression of proliferation-associated genes as well as several collagen genes. The investigators subsequently used supervised principal components analysis specifically to develop a set of 259 genes with prognostic relevance. In an independent validation group, this gene set strongly predicted survival and was independent of tumor stage, grade, and performance status. The authors note that their 259 gene set contained 15 of the 51 genes that had previously been published by Takahashi.

Yao et al. compared 28 clear cell RCC with 9 normal kidney samples, 3 chromophobe carcinomas, 1 cystic clear cell RCC, and 1 adult Wilms' tumor. Not only were the clear cell carcinomas distinct from the other samples by hierarchical clustering, the clear cell tumors clustered into 2 groups with different outcomes. One cluster contained the high-grade and/or high-stage carcinomas, whereas the other contained low-grade and/or low-stage carcinomas. Six of 13 patients in the aggressive cluster died of their disease, whereas only 3 of 13 died in the less-aggressive cluster. This group then pursued 2 genes, ADFP and NNMT, by RT-PCR on a larger group of tumors. ADFP was additionally investigated by immunohistochemistry. They were able to confirm a statistically significant association with clear cell RCC and particularly with the good prognosis subgroup. High expression of ADFP was significantly associated with a better prognosis.

Other groups have attempted to identify gene expression patterns that characterize aggressive clear cell RCC using metastatic samples. Kosari et al.[38] studied 9 primary aggressive clear cell RCC, 10 primary nonaggressive clear cell RCC, and 9 metastatic clear cell RCC together with 12 non-neoplastic kidney samples. Aggressive cases were defined as those that resulted in death or metastasis within 4 years of nephrectomy. They found that the expression patterns of aggressive primary clear cell RCC were similar to metastatic clear cell RCC, but unsupervised hierarchical clustering permitted separation of most of the aggressive cases and metastases from primary nonaggressive clear cell RCC. They identified a set of 35 genes that permitted discrimination between these groups and validated the results using quantitative RT-PCR on a separate group of cases. Among this set, they identified a single gene, survivin (BIRC5), that they evaluated at the protein level by immunohistochemistry on a separate and independent set of 183 clear cell RCC. Expression of survivin was inversely correlated with survival in multivariate analysis.

A slightly different emphasis was used by Jones et al. in a study that stressed the differences between clear cell RCC of differing stage and particularly those that had metastases[24]. These investigators studied 22 primary clear cell RCC and 10 metastatic clear cell RCC. By evaluating normal kidney, primary tumors, and metastases, they were able to identify 31 genes that showed progressively increasing or decreasing expression across the normal to primary tumor to metastasis spectrum. Eight of these genes showed increasing expression with tumor progression and 13 showed decreasing expression with tumor progression. A group of 155 genes was identified that were overexpressed in metastases relative to primary tumors that had not developed metastases. This group of genes was differentially expressed in the 9 independent cases that had or later developed metastases such that all 4 cases that showed a metastatic pattern of gene expression had metastases at the time of nephrectomy whereas 4 of the 5 that showed an expression pattern more similar to the $T_1$ cases were free of metastasis at the time of nephrectomy. They then validated this list of genes on an independent data set published by Sültmann et al.[20]. Using 41 genes from the set that were on the Sültmann arrays, unsupervised hierarchical clustering separated the Sültmann cases into 2 groups. One of these contained 13 cases that were $M_1$ at presentation and 6 cases that were $M_0$, whereas the other cluster contained 11 cases that were $M_0$ and 6 that were $M_1$ ($p < 0.05$). Finally, they clustered their cases using 123 of the 128 genes identified by Ramaswamy et al.[39] as a global metastasis signature. As had been demonstrated by Ramaswamy et al. on a range of different epithelial tumors, this gene list permitted separation of distant metastatic tumors and tumors with metastasis at the time of surgery from most of the $T_1$ tumors and from the $T_2$ and $T_3$ tumors that had not developed metastases at the time of surgery.

The prognostic genes identified by all of these researchers are similar in that the prognostic genes were predominantly more highly expressed in good prognosis cancers and less highly expressed in the poor prognosis cancers. This may be because the poor prognosis tumors are less well differentiated in general and are less likely to express genes associated with differentiation. If this is true, then a large number of genes may need to be investigated before one or more are discovered that are pathogenetically involved in the progression or RCC.

## Comparisons Between Studies

Much has been made of the fact that different studies have reported different clusters of "renal cell carcinoma genes"[25]. This has been construed as a weakness of gene array data; however, it should really be recognized as an intrinsic feature of microarray data analysis. Of course, individual genes are of critical importance; however, the great excess of genes analyzed relative to the number of samples surveyed represents a form of multiple hypothesis testing. In other words, genes that appear to be associated with a particular class of sample must be expected to contain at least some genes that show apparent specificity as a result of random variation rather than true class association. It is unlikely that a second independent study will rediscover these same genes. This highlights the importance of unsupervised clustering in the analysis of microarray data. Given two classes of sample and 40,000 or more genes to distinguish between the two, a number of genes that show an apparently specific association with either class will be found. What is striking is if this population of 40,000 genes can blindly identify the two classes of tumor without any prior knowledge of the class assignment. With such an unsupervised method, some of the genes associated with each class will certainly be associated due only to random chance, but some should truly indicate a biological difference between the samples.

There are other causes of this apparent lack of reproducibility. Genes are represented on an array by segments of DNA that are classified by GenBank accession number. These are assembled into Unigene clusters, which can be defined as "genes". Typically, each gene on an array is represented by several different probesets or GenBank accession numbers. As noted by Lenburg et al.[25], a conservative method of identifying genes overexpressed in RCC would be to accept only those genes for which all of the different spots provide similar evidence of overexpression. These authors found that this approach was too conservative and they instead elected to average the expression for each gene (Unigene cluster) across all of the probesets that represent it. In addition to adding their own data to the literature, Lenburg et al. compared their data to several previously published datasets. They identified 113 genes for which 3 or more studies showed differential expression in RCC vs. normal kidney. Their analysis makes clear that genes such as IGFBP3, VEGF, FBP1, LOX, KL, PLOD2, CXCR2, and vimentin have repeatedly been found to be overexpressed in clear cell RCC.

Another problem with comparison of data between different publications is that the study designs differ. Not only do array platforms vary, but the type of common reference may vary from patient-matched normal kidney, to pooled normal kidney, to pooled tumor cell line. The samples studied sometimes represent tumor cell lines that are known to have dramatically different phenotypes from fresh tissue. Furthermore, the samples may be characterized only as "renal cell carcinoma" rather than a specific subtype. Some studies have been performed with the goal of identifying prognostic markers, others with a goal of identifying useful markers for class distinction. These differences introduce subtle variations in the genes ultimately found to be associated with prognosis or tumor type. Certainly a direct coclustering of the datasets is complicated by a difference in common reference. However, insight into the validity of a gene set identified in one paper may certainly be gained by surveying those particular genes on a second sample set.

One aspect of comparison between studies that must be stressed is the importance of public availability of data. Obviously an essential component of microarray technology is the massive amount of data generated with relatively few samples. It is not possible to publish this data by conventional means and, as a result, most publications list fewer than a hundred genes even though tens of thousands have been studied. The published gene lists alone are of little to no use to the scientific community and clearly fail to do justice to the scope of the data that lie behind the publication. Publicly available data sets can be and are used to permit reanalysis of data and discovery of aspects of disease. For example, Jones et al.[24] were able to use the Higgins[28] publicly available data set to validate their findings for renal tumor class prediction and the Sültmann[20] publicly available data set to validate their metastasis gene signature. Similarly, Lenburg et al.[20] were able to reanalyze the Higgins data in order to achieve a completely different, but fully valid, emphasis of the data than had been intended by the original authors.

The clear cell RCC series published by Zhao et al.[37] may be used to illustrate the possibilities for reanalysis of a publicly available data set. This data set, like all published data sets curated on the Stanford Microarray Database http://genome-www.stanford.edu/microarray, can be easily downloaded for analysis. It is then possible to evaluate all of their cases or a subset of cases of particular interest. For example, those patients with no evidence of disease at greater than 5 years of follow-up and patients who either died of disease or are alive with disease after nephrectomy represent 124 cases. It is possible to evaluate these cases for expression of previously published gene sets of biological interest; for example, the metastasis signature published by Jones et al.[24], the global metastasis signature of Ramaswamy et al.[39], and the aggressive genes published by Kosari et al.[38]. One finds that 50 of the 155 gene metastasis signature of Jones are present and well measured on the Zhao array. Similarly, 29 of the 128 genes published by Ramaswamy and 9 of the 34 published by Kosari are present and well measured. If one performs Significance Analysis of Microarrays (SAM)[40] analysis of these genes, one can rank order these genes according to the strength of their association with patients who developed metastasis. By this analysis, the top 10 genes associated with aggressive behavior in the Zhao data set are: KPNB1 (Jones), BIRC5 (Kosari), NMT1 (Jones), TRA1 (Jones), HTATSF1 (Jones), COL1A2 (Ramaswamy), TIP120A (Ramaswamy), CKS2 (Kosari), FXR1 (Jones), and CSPG6 (Jones). It is therefore possible to validate one set of published aggressive genes on an independent data set.

## ARRAY BASED COMPARATIVE GENOMIC HYBRIDIZATION

Sültmann et al.[20] were able to correlate their gene expression analysis with karyotypic abnormalities determined by classical cytogenetics and found that copy number changes do influence expression in RCC. However, given the acknowledged importance of cytogenetic changes in the classification of adult renal tumors, surprisingly little work has been done on array-based comparative genomic hybridization. This may be because array CGH is newer and more technically demanding than expression array research. Another factor may be that investigators are less comfortable with methods of data analysis and particularly, the difficulties of analyzing data using hierarchical clustering. Wilhelm et al.[41] used array-based comparative genomic hybridization to classify renal tumors. They were able to correctly classify 33 of 34 tumors using only data based on copy number changes identified by array CGH. Valle et al.[42] used array CGH in an attempt to identify microdeletions in 3p in three cases of familial ccRCC that lacked VHL mutations. These investigators hoped to identify other loci involved in the pathogenesis of clear cell renal carcinoma. However, they failed to identify any such loci. Finally, Yang et al.[33] used comparative genomic microarray analysis, a technique which predicts copy number changes based on gene expression, to infer cytogenetic alterations in papillary RCC.

The promise of high-level resolution of copy number changes in renal tumors should lead to future array CGH studies of renal tumors.

## CLINICALLY RELEVANT ADVANCES

It will take time to translate the massive amount of data generated by microarray experiments into clinically relevant advances. Promising findings need first to be validated on independent patient groups and more laborious techniques of molecular biology may be necessary to fully characterize the behavior of individual genes. Because of the very large number of genes interrogated in array studies, the behavior of interesting genes needs to be validated by real-time RT-PCR, *in situ* hybridization, or immunohistochemistry. For many of the existing studies, such validation remains to be done. It may be necessary to begin new studies, for example, to detect serum markers, from the point of sample collection. The majority of interesting genes will fail to prove useful for clinical practice for a variety of reasons. It would be wise to anticipate that the performance and analysis of the microarray experiment represents only the beginning and minor component of the effort to improve clinical patient care.

Nevertheless, early advances have certainly already been made. Diagnostic markers for use in immunohistochemistry have been discovered. These include vimentin in RCC[17,28,43], AMACR in papillary RCC[44], and defensin-1 and parvalbumin in chromophobe carcinoma and oncocytoma[43]. Some of these markers are currently used in diagnostic surgical pathology. These markers certainly represent only a small fraction of those that have been investigated and found for a variety of reasons to be of no clinical value. Yet, they attest to the validity of microarray data. Immunohistochemical markers that stratify patients according to prognosis, such as survivin, clearly will require more sophisticated study than those that simply permit class distinction. But there is every reason to believe that such markers will be discovered and prove useful. The same is true for serum markers that may permit early detection of renal carcinoma. This assessment should not serve to discourage expression array research, but only to highlight the formidable amount of work necessary to translate the tremendous promise of gene array studies into advances in clinical patient care.
